# Study of Allowable Interlaminar Normal Stress Based on the Time–Temperature Equivalence Principle in Automated Fiber Placement Process

**DOI:** 10.3390/polym13234180

**Published:** 2021-11-29

**Authors:** Rui Xiao, Jiaqi Shi, Jun Xiao

**Affiliations:** College of Material Science and Technology, Nanjing University of Aeronautics and Astronautics, Nanjing 210016, China; jiaqi_shi@nuaa.edu.cn (J.S.); j.xiao@nuaa.edu.cn (J.X.)

**Keywords:** composite material, automated fiber placement, strength, stress, tackiness

## Abstract

Automatic fiber placement (AFP) is a type of labor-saving automatic technology for forming composite materials that are widely used in aviation and other fields. In this process, concave surface delamination is a common defect, as existing research on the conditions for this defect to occur is insufficient. To predict the occurrence of this defect, the concept of allowable interlaminar normal stress is proposed to define its occurrence conditions, and based on this concept, probe tests are carried out using the principle of time–temperature equivalence. Through the laying speed/allowable normal stress curve measured in the probe experiment, the physical meaning of allowable normal stress is discussed. At the same time, the measured curve is quantitatively analyzed, combined with viscoelastic theory and the molecular diffusion reptation model, and the dominating effect in the formation of a metal/prepreg layer and prepreg/prepreg layer is determined. Finally, the experimental data are used to guide the parameter selection in an automatic placement engineering case and prove its correctness.

## 1. Introduction

**Figure 1:**
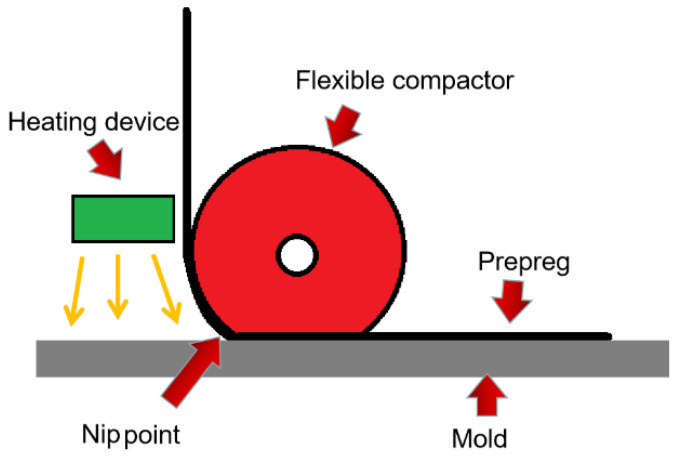
ThermosetAFP working process.

Automatic fiber placement (AFP) technology is a production process in which resin-impregnated carbon fiber tape (prepreg) is automatically laid on a mold to make composite parts. This process has been widely used in the aviation industry in the past 30 years [1]. Figure 1 shows the working process of a thermoset AFP head. Compared with automatic tape laying (ATL) technology, AFP is more adaptable and can be used to manufacture composite material parts with complex curved surfaces [2].

However, in actual production, the AFP process can only handle complex geometries to a certain degree. For example, fiber tension produces a component in the normal direction of the concave surface, which causes the prepreg to fall off the laminate surface [3]. There are two sources of fiber tension. To ensure the reliability of AFP equipment, the fibers need to maintain a stable process tension. Additionally, since each prepreg has a certain width (e.g., “1/4”, “1/2”), additional tension will also be generated if the fiber trajectory steers in the plane of the prepreg [4]. Since the bonding between the prepreg and the mold surface is weaker than the bonding between prepregs, this problem is particularly serious when the first layer is laid. For this reason, it is necessary to study the correlation law of prepreg bonding strength and process parameters.

In existing research, the ability of prepregs to form bond strength is often referred to as tack. The first step in studying the tack of prepregs is to accurately measure it. Common tack test methods mainly include the probe test and the peel test [5]. Both tests are usually carried out on a universal testing machine. The method is to separate the sample after the bonding is formed and record the displacement and separation force during the process. The difference between the two methods is that in the probe test, the sample is stressed and falls off in the direction perpendicular to the joint surface. In the peel test, the stress point of the sample is at its edge, and the sample gradually peels off along the joint surface. In existing research, the probe test mainly measures the maximum normal stress that the sample can withstand during the separation process [6], and the peel test mainly measures the average peeling force during the peeling process. There are many forms of peeling tests, among which the experimental methods developed by Crossley, Schubel, and Warrior are particularly suitable for simulating the AFP process [7]. According to Williams and Kauzlarich’s analysis, the peel force per unit width of the sample represents the interfacial energy per unit area of the laminate [8].

The process parameters that need to be controlled during the AFP process include laying temperature, laying speed, and laying pressure. The above three process parameters all affect the prepreg’s bonding strength. Laying temperature is a key factor affecting the tack of the prepreg [9]. It refers to the temperature at the jointing point of the prepreg raw material and the existing ply—that is, the temperature at the nip point in Figure 1. AFP equipment usually has a heating device to increase laying temperature and facilitate bonding. The suitable laying temperature in industrial production is mainly obtained by the trial-and-error method [10], and the laying temperature of thermosetting prepreg is usually around 40 °C. Ahn et al. [11] found that the tack of the prepreg exhibits a bell-shaped curve with temperature, and the maximum point of tack is 20–25 °C above the glass transition temperature of the prepreg resin; this is also consistent with actual production experience. The laying speed usually refers to the linear speed at which a single prepreg is placed on the surface of the product. It is determined by the speed of the equipment. In order to improve productivity, the laying speed of modern AFP machines can reach more than 1000 mm/s. Since the diameter of the flexible compactor that applies the laying pressure is usually about 60 mm, the chord length in contact with the mold when it is deformed is about 10 to 20 mm. This means the pressure time of the laying process is only a few milliseconds. [12] The biggest challenge in measuring the bonding strength of the AFP process is to reproduce this ultra-short pressure process with experimental means. Crossley et al. [13] found that the time–temperature equivalent coefficient measured by the rheological behavior of the prepreg can be used to predict the viscosity performance of the prepreg. According to this conclusion, the low-temperature and low-speed experiments can be used to simulate the actual high-temperature and high-speed AFP process. Smith et al. [14] and Endruweit et al. [15] used this method to estimate the bonding strength of the AFP process through peeling experiments, and the measured maximum placement speed can reach 2500 mm/min. Budelmann et al. [16] found through probe tests that the bonding strength between prepreg layers increases with the increase in pressure time. Laying pressure refers to the pressure of the flexible compactor on the prepreg ply. The compactor pressing force of the AFP process is usually 1 N–5 N per millimeter of the compactor length. Dubois [6] found that the bonding strength between prepreg layers increases with the increase in laying pressure. Endruweit et al. [17] found that when the laying pressure is small, the bonding strength between layers increases rapidly with the increase in pressure, but when the laying pressure is high, the change in the bonding strength between layers with pressure is relatively insignificant.

Although the above research has improved the understanding of the formation mechanism of interlayer bonding strength, applying their conclusions to solve specific process problems remains difficult for multiple reasons. Firstly, although the time–temperature equivalent/peeling test method can be used to measure the peeling force at the actual laying speed, the occurrence of prepreg delamination mainly depends on the normal stress that the joint surface can withstand, which is not consistent with peeling force in the physical meaning. Second, the existing probe test method can measure the maximum tensile strength when the ply is separated. When the maximum tensile force is reached, however, the distance between the two layers of prepreg can reach 0.1 mm to 0.5 mm [18]. Since the thickness of one layer of prepreg is only 0.1 mm to 0.2 mm, this distance means that the delamination defect between the layers has already occurred long in advance, which reduces the practical significance of the test result. Practical experience has proved that, if the maximum separation force measured by the probe test is used to predict the occurrence of delamination defects, the probability of defects occurring will be seriously underestimated. Third, the current research has not studied in detail the dominant physical effects in the interface bonding process. The analysis of process parameters remains mostly at the stage of qualitative analysis of discrete points, and the mathematical laws have not yet been summarized. Therefore, existing research is insufficient to predict the occurrence conditions of the debonding phenomenon on the concave surface.

In order to measure the critical condition of prepreg delamination, this research proposes the concept of allowable interlaminar normal stress. The allowable interlaminar normal stress is the normal stress when normal tension is applied to the ply to allow its thickness to reach the thickness of the raw material, denoted as $\sigma_{T}$. This study will use the principle of time–temperature equivalence to conduct probe tests at lower temperatures to simulate the actual high-temperature and high-speed AFP process, thereby predicting $\sigma_{T}$ under different working conditions. At the same time, this study will combine the reptation model and viscoelastic mechanics theory to study the law of change $\sigma_{T}$ under different laying speeds and determine the dominant physical effect of the bonding process. As a result, engineers can use parameters such as mold curvature and trajectory radius to obtain the allowable normal stress at each point on the trajectory and select an appropriate layup speed and prepreg tension according to the relationship between the layup speed and $\sigma_{T}$.

## 2. Related Physical Model

### 2.1. Mechanical Model of Debonding Phenomenon on Concave Surface

**Figure 2:**
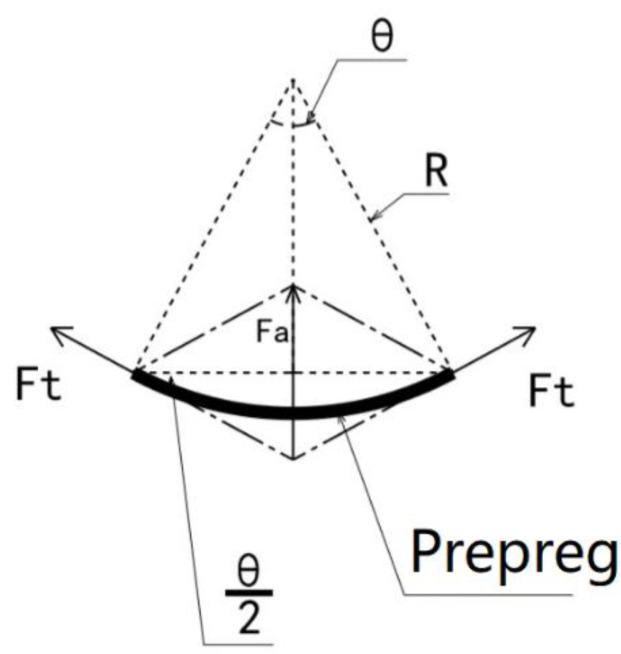
Delamination mechanism of prepreg on the concave surface.

According to the mechanics and calculus theory, the fiber tension along the tangential direction of the negative curvature surface will produce a normal stress component in the normal direction of the surface, the magnitude of which is inversely proportional to the radius of curvature; see Figure 2.

Figure 2 shows a prepreg microelement on a concave curved surface. Let the microelement length be dl, the radius of the curved surface be R, and the fiber tension be $F_{t}$. It can be seen that $F_{t}$ will produce a component force $F_{a}$ on the normal side of the surface. The magnitude is:

(1) $$F_{a}=2\cdot \sin(\frac{\theta }{2})\cdot F_{t}=\theta \cdot F_{t}=\frac{dl}{R}\cdot F_{t}$$

Therefore, the fiber tension will produce normal stress $\sigma_{a}$ on the surface of the prepreg. Setting the fiber width as W, we obtain:

(2) $$\sigma_{a}=\frac{F_{a}}{dl\cdot W}=\frac{\frac{dl}{R}\cdot F_{t}}{dl\cdot W}=\frac{F_{t}}{R\cdot W}$$

Equation (2) shows that the possibility of bridging at each point on the fiber path depends on the fiber tension and the path curvature at that point. Due to the non-linear tack of the prepreg, the occurrence of bridging is a probability problem. Under the same radius of curvature, when the turning angle θ increases, the fiber length will increase, and the probability of bridging will increase accordingly. However, this is a global problem. From the process parameter point of view, the purpose of this research is to find the local critical point and apply the corresponding process parameters to the full path to minimize the probability of bridging. Therefore, Formula (2) is used as the starting point for the research. It can be seen from the above formula that when $\sigma_{a}\geq \sigma_{T}$, the prepreg will be debonded; the greater the fiber tension, the smaller the radius of curvature, and the easier it is for defects to occur.

### 2.2. Time–Temperature Equivalence Principle

The principle of time–temperature equivalence means that the same viscoelastic behavior of a polymer can be observed at a higher temperature for a shorter period of time, or it can be observed at a lower temperature for a longer period of time. Therefore, it is possible to study the viscoelastic performance of the polymer in a short time under high temperatures with a longer action time at a low temperature. Crossley [13] found that by testing the rheological behavior of the prepreg resin, the time–temperature equivalent parameters can be obtained in the form of the Williams–Landel–Ferry (WLF) equation. The viscoelastic behaviors of the prepreg predicted by these parameters, such as the bonding strength and compressive creep of the prepreg, are highly consistent with the experimental measurement results. This shows that the bonding strength of the prepreg conforms to the principle of time–temperature equivalence and can be predicted by the time–temperature equivalent coefficient measured in the creep behavior. Suppose the time–temperature conversion coefficient from temperature $T_{0}$ to temperature T is $\alpha_{T}$, and $C_{1}$/$C_{2}$ are constants that can be calculated from experimental data ($C_{1}$ is dimensionless, $C_{2}$ is in the unit of K), then the form of the WLF equation is:

(3) $$\log(\alpha_{T})=\frac{-C_{1}(T-T_{0})}{C_{2}+(T-T_{0})}$$

Let $D_{T_{0}}(t_{0})$ be the viscoelastic behavior quantity of the prepreg with the action time $t_{0}$ when the temperature is $T_{0}$; then, at any temperature T, the viscoelastic behavior quantity $D_{T}(t)$ of the prepreg with the action time t can be calculated as follows:

(4) $$D_{T}(t)=D_{T_{0}}(\frac{t}{\alpha_{T}})$$

Gergesova et al. [17] give a closed-form solution method for calculating the time–temperature conversion coefficient $\alpha_{T}$ according to the viscoelastic behavior curve at different temperatures. See reference [18] for the specific calculation method.

### 2.3. Prepreg Interlayer Bonding Model

**Figure 3:**
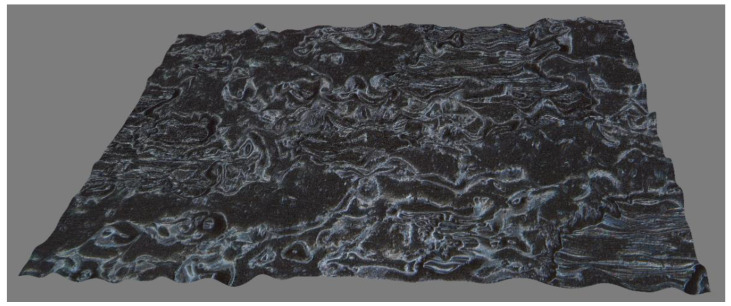
Surface morphology of M21C prepreg.

Wool and O’Connor [18] described the resin interface fusion process. Let the glass transition temperature of the resin be $T_{g}$; when $T\geq T_{g}$, the process can be divided into five stages, namely, surface rearrangement, surface approach, wetting, diffusion, and randomization. There are two dominant effects in this process. The first is the increase in resin contact area caused by the deformation of the resin. The second is that when the resin–resin interface is formed, the interpenetration of molecules on both sides of the interface leads to an increase in interface bonding strength. For prepregs, however, the situation is different from pure resin bonding. Figure 3 shows the surface morphology of Hexcel M21C prepreg taken with a Leica DVM 6A three-dimensional profiler. It is found through measurement that resin is randomly distributed on the surface of the prepreg in blocks, and its height ranges from about 30 to 40 microns. When the prepreg is compressed, these resin asperities will continue to deform, resulting in a continuous increase in the interface resin contact area. At the same time, for the resin–resin interface that has been in contact, the resin molecules on both sides of the interface will continue to diffuse until the strength of the interface reaches the strength of the resin matrix. In other words, during the bonding process, for each specific resin–resin interface, the bonding process may be in one of the above five stages. However, for the whole piece of prepreg, due to a large number of resin asperities and their different shapes, these five stages may occur simultaneously on different resin asperities.

Aiming at the resin deformation in the above two dominant effects, Lee and Springer [19] used laminar flow theory to simplify the resin deformation process and came to the following conclusions:

(5) $$D_{ic}=g^{*}\cdot {\left[\int_{0}^{t}\frac{P_{app}}{\eta_{mf}}dt\right]}^{1/5}$$

In the above formula, $D_{ic}$ is the close contact rate of the interface resin, $g^{*}$ is a constant determined by the geometric distribution of the interface resin, and t is the time of applying pressure. $P_{app}$ is the pressure applied to the resin, and $\eta_{mf}$ is the viscosity of the resin system. The main problem of Equation (5) is that its accuracy has a lot to do with the parameter $g^{*}$, which represents the geometric characteristics of the prepreg surface and is difficult to measure [20]. Loos et al. [21], aiming at removing any empirical data from the model, used a surface analyzer to characterize the surface roughness or waviness of APC-2 prepreg sheets and determine the value of $g^{*}$. However, Bulter et al. [22] pointed out that the measurements given by current profilometry methods or deduced from micrographs of the actual surface were not consistent with the model as they give information of the real shape of the surface and not the rectangular shape. However, assuming $t_{c}$ is the time required for the resin to fill the entire interface, it still can be seen that if the pressure does not change during the laying process, when $t\leq t_{c}$, there are:

(6) $$D_{ic}\propto t^{1/5}$$

Aiming at molecular diffusion, Wool et al. [19] used the reptation model to establish the relationship between the interface strength $\sigma_{i}$ and the diffusion time $t_{d}$. Letting $t_{r}$ be the time for the molecules on both sides of the interface to diffuse completely so that the strength of the interface no longer increases, $\sigma_{\infty }$ is the strength of the resin matrix, M is the molecular weight of the resin, when $t_{d}\leq t_{r}$:

(7) $$\frac{\sigma_{i}}{\sigma_{\infty }}\propto t_{d}{}^{1/4}\cdot M^{-3/4}$$

When $t_{d}>t_{r}$:

(8) $$\frac{\sigma }{\sigma_{\infty }}\propto M^{-3/4}$$

For certain types of prepregs, M is a fixed value. Therefore, when $t_{d}\leq t_{r}$:

(9) $$\sigma_{i}\propto t_{d}{}^{1/4}$$

If delamination defects occur under the allowable normal stress $\sigma_{T}$, it is obvious that $\sigma_{T}\propto D_{ic}\cdot \sigma_{i}$. It should be noted that Formula (6) describes the deformation process of the entire prepreg surface after being compressed, while Formula (9) describes the local phenomenon when a pair of specific resin asperities are in contact. Since contact is a prerequisite for diffusion, and as mentioned above, the surface morphology of the prepreg is complex, and the time when the resin asperities start to contact varies from place to place, so $t_{d}$ is not equal to t.

For the prepreg/metal interface, the resin molecules cannot diffuse into the metal side; that is, $\sigma_{i}$ is a constant. Therefore, if the logarithm of $\sigma_{T}$ and the laying speed V are taken at the same time and the $\log(V)-\log(\sigma_{T})$ curve can be fitted with a straight line with a slope of −1/5, then the correctness of Formula (6) can be verified, and the relationship between laying speed and allowable normal stress can be determined.

For the prepreg/prepreg interface, if the resin asperities on the two layers of prepreg all come into contact at the moment of pressure and the molecular diffusion is always going on during the entire laying process, then $t_{d}$ is equal to t. In this case, $\sigma_{T}\propto t^{\frac{1}{5}}\cdot t^{\frac{1}{4}}=t^{0.45}$. Therefore, the slope of the $\log(V)-\log(\sigma_{T})$ curve should be −0.45. On the other hand, if the slope of $\log(V)-\log(\sigma_{T})$ is −0.2 (consistent with the prepreg/metal interface), it proves that $\sigma_{i}$ can be regarded as a constant. This shows that the time $t_{r}$ for complete molecular diffusion is so short that the effect of molecular diffusion on the bonding strength can be completely ignored. Additionally, if the slope of $\log(V)-\log(\sigma_{T})$ is between −0.2 and −0.45, it means that the resin asperities on the two layers of prepreg are gradually in contact, and the allowable normal stress is affected by both resin deformation and molecular diffusion. 

## 3. Design of Experiments

In order to simulate the actual AFP process, all of the following experiments were carried out using the M21C prepreg produced by Hexcel, which is commonly used in automatic laying.

### 3.1. Design of Time–Temperature Equivalent Coefficient Measurement Experiment

**Figure 4:**
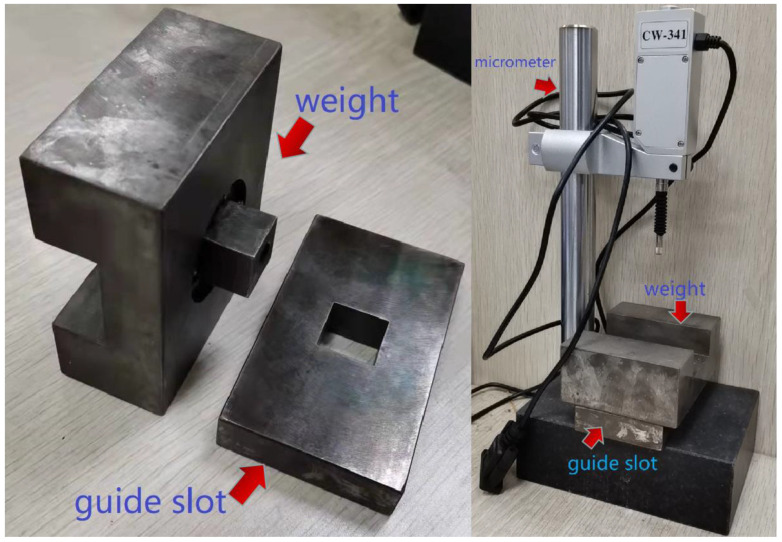
Prepreg creep curve measuring device.

**Figure 5:**
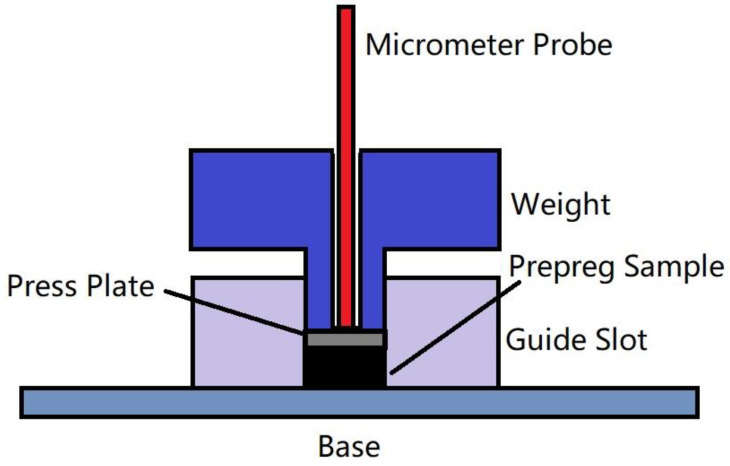
Scheme of prepreg creep curve measuring device.

As mentioned above, the optimal temperature for automatic placement is around 40 °C, and the time–temperature equivalent coefficient measured by the viscoelastic properties of the prepreg can be used to predict its viscosity performance. Therefore, by repeating the pressure creep experiment of the prepreg at 40 °C and normal temperature, and comparing the creep curves of the two, the time–temperature conversion coefficient of the prepreg from room temperature to laying temperature can be calculated. The experimental device is shown in Figure 4 and Figure 5.

In this experiment, the creep curves of prepreg samples under compression were measured in an environmental chamber at 40 °C and room temperature at 22 °C. The experimental pressure is applied by a weight, which is constrained by the square hole of the guide slot to ensure that the pressure on the sample is vertical and uniform. The prepreg sample is placed in the square hole of the guide slot; its size is 20 mm × 20 mm (the same as the square hole) and it is made of 10 layers of prepreg in the same direction. Before the experiment, the sample was compressed with the weight of this experiment for 10 min to eliminate the influence of uneven stacking. A press plate with the same size as the square hole is placed on top of the prepreg sample, which is compressed by the gravity of the weight and press plate. Using a micrometer (the probe of which goes through a hole in the weight and contacts the press plate) to measure the displacement of the press plate, the prepreg creep curve can be obtained, and the time–temperature equivalent coefficient can be calculated according to the method used by Gergesova et al. [17].

### 3.2. Design of Allowable Normal Stress Measurement Experiment

The allowable normal stress measurement method is as follows: use a universal testing machine to apply pressure to the prepreg sample with the pressure and time equivalent to the actual AFP process. Then, separate at a constant speed and measure the displacement and separation force in real-time. When the sample is separated to exceed the original ply thickness before being compacted, the measured separation normal stress shall be the allowable normal stress $\sigma_{T}$. By changing the applied pressure and pressing time, the equivalent laying speed/allowable normal stress curve can be measured under different laying pressures. Due to the high speed of the AFP process, if the experiment is carried out directly under the actual laying condition of 40 °C, the pressure holding time will be too short. This exceeds the control range of the universal testing machine. Therefore, this experiment uses the measured time–temperature equivalent coefficient to perform equally slowly at a room temperature of 22 °C.

**Figure 6:**
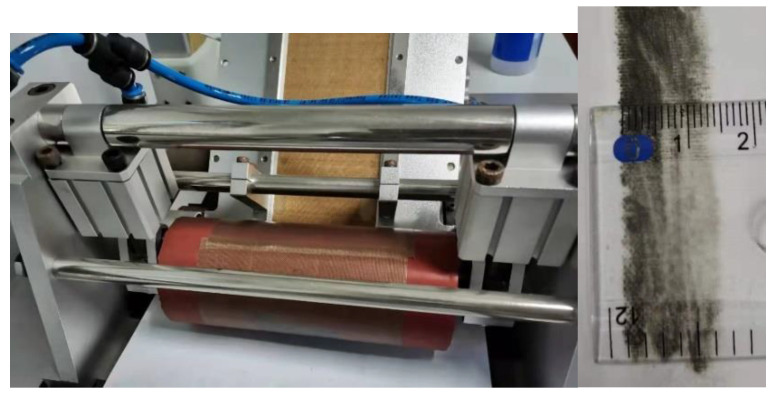
Measurement experiment of contact length of the flexible compactor.

In the actual AFP process, laying pressure is not uniformly distributed along the contact length between the compactor and substrate. However, the constant average pressure is used to represent actual laying pressure in the probe test to simplify the test design. In order to make the probe test pressure close to the actual laying parameters, it is first necessary to measure the average pressure applied by the AFP machine to the prepreg during the laying process. Figure 6 shows a simulation device for the flexible compactor of an AFP machine with adjustable compression force. The measurement experiment method is to coat the compactor with pigment and press it onto the paper under different laying pressures. By measuring the indentation width h (unit: mm) on the paper, the average pressure P (unit: kPa) under the laying pressure can be obtained, as shown in Figure 6:

After the average laying pressure P and the indentation, width h is obtained, the pressure holding time $t_{p}$ (unit: s) and pressure holding force F (unit: N) of the probe test can be equivalently converted:

(10) $$t_{p}=\frac{h}{V\cdot \alpha_{T}}$$

(11) F=P·S

**Figure 7:**
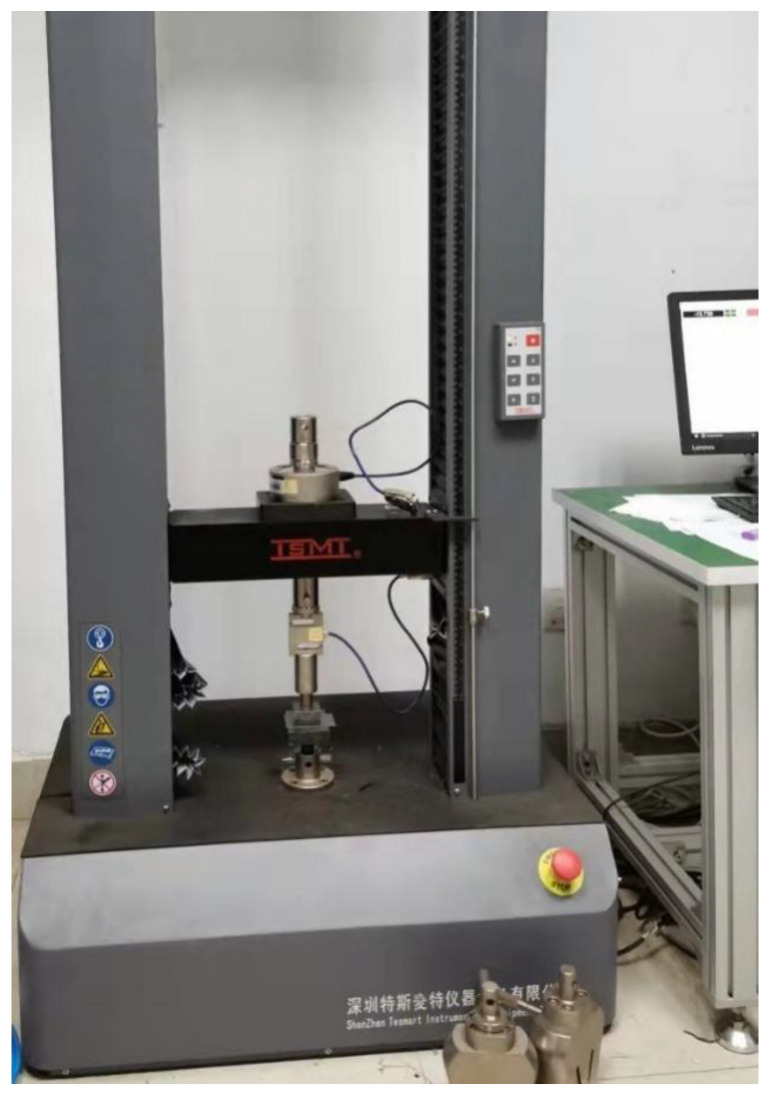
Universal testing machine and test probe.

In the above two equations, V is the laying speed (unit: mm/S), $\alpha_{T}$ is the time–temperature equivalent coefficient, and S is the probe area (unit: $m^{2}$). This experiment measures the $\sigma_{T}$ of prepreg/steel and prepreg/prepreg ply when the compactor pressing force per millimeter of the compactor length is 1 N, 3 N, and 5 N (corresponding values P in the unit of kPa are shown in Section 4.2.1), and the laying speed is 30, 60, 100, 150, 200, 300, and 400 mm/s. Figure 7 shows the universal testing machine and experimental probe. The dimension of the contact surface of the probe is 30 mm × 30 mm. Therefore, $S=0.009\;m^{2}$. The universal testing machine used in the probe test is the model EM6. 204-L, manufactured by ShenZhen Tesmart Instrument and Equipment Co., Ltd. The load cell used is the model BSA-XS-50 kgP, manufactured by Transcell, shown in Figure 7. 

## 4. Test Results

### 4.1. Time–Temperature Equivalent Coefficient Measurement Results

**Figure 8:**
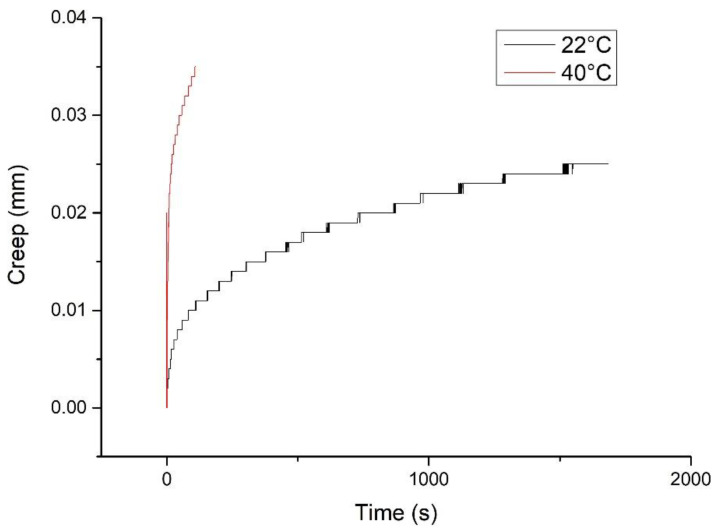
The 22 °C and 40 °C compressive creep curve of the prepreg sample.

Figure 8 shows the creep curve of the prepreg sample measured at 22 °C and 40 °C respectively:

Using the method of reference [18] to calculate the time–temperature equivalent coefficient in Equation (4), it can be found that when $T_{0}=22^{\;o}C$ and $T=40^{o}C$,$\alpha_{T}=0.0104$.

### 4.2. Allowable Normal Stress Measurement Results

#### 4.2.1. Compactor Deformation Measurement Results

The measurement shows that when the compactor pressing force is 1 N/mm, the indentation width is 10 mm; that is, the actual average laying pressure is 100 kPa. When the compactor pressing force is 3 N/mm, the indentation width is 14 mm; that is, the actual average laying pressure is 214.3 kPa. When the compactor pressing force is 5 N/mm, the indentation width is 18 mm; that is, the actual average laying pressure is 277.8 kPa. Incorporating parameters such as probe area S and time–temperature equivalent coefficient $\alpha_{T}$ into Equations (10) and (11), it can be seen that the experimental conditions of the probe test are as follows:

When the laying pressure is 100 kPa, the pressure holding force F of the universal testing machine is 90 N, and the holding time $t_{p}$ corresponding to different laying speeds is shown in Table 1.

When the laying pressure is 214.3 kPa, the pressure holding force F of the universal testing machine is 192.87 N, and the holding time $t_{p}$ corresponding to different laying speeds is shown in Table 2.

When the laying pressure is 277.8 kPa, the pressure holding force F of the universal testing machine is 250.02 N, and the holding time $t_{p}$ corresponding to different laying speeds is shown in Table 3.

#### 4.2.2. Allowable Normal Stress Measurement Result of Prepreg/Metal Laminate

**Figure 9:**
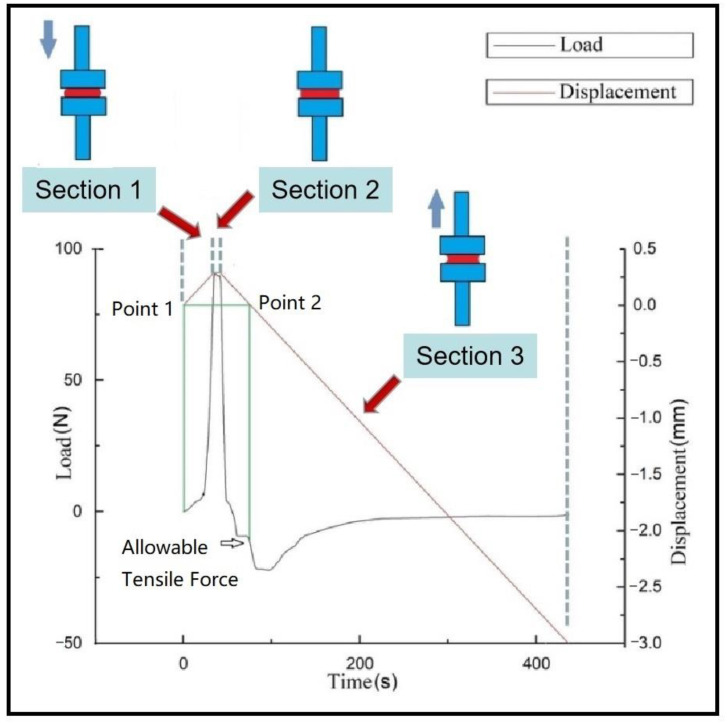
Example of probe test curve.

This probe experiment measures the time/load curve and time/displacement curve between the prepreg/metal laminate during the pressurization–holding–separation process. At the beginning of the experiment, the prepreg is glued to the upper probe, and the lower probe is a metal surface. Each test process can be divided into three sections. The first is the pressurization section. In this section, the upper probe moves downward. When the upper probe is in contact with the lower probe surface, the pressure is first increased. In this section, the thickness of the sample decreases, and the pressure increases. At this time, the displacement and load measured by the universal testing machine both increase in the positive direction. The second section is the pressure-holding section. After the load reaches the required pressure holding force F calculated in Section 4.2.1, the displacement is maintained for a period of time. In this section, the measured displacement and load remain unchanged. The third section is the separation section. After the pressure is maintained for the equivalent holding time $t_{p}$ calculated in Section 4.2.1, the upper probe moves upward at a constant speed, and the experiment enters the separation section. In this section, both the displacement and the load move in the negative direction. When the load is positive, it means that the sample is still under pressure. When it is negative, it means that the sample is under tension. When the displacement is positive, it means that the thickness of the sample is still less than the thickness of the raw material. When it is negative, it means that the thickness of the sample is greater than the thickness of the raw material. According to the definition of allowable normal stress $\sigma_{T}$, when the displacement returns to 0, we record the allowable tensile force $F_{s}$ (in N). An example of the measured curve is shown in Figure 9. The green lines in the figure are auxiliary. The lower end of the leftmost vertical green line is the load zero point, so the corresponding displacement—that is, the intersection point of the green line and the displacement curve—represents the original thickness of the sample. Point 2, where the horizontal green line from point 1 crosses the displacement curve, represents the point at which the sample returns to the thickness of the raw material during the separation phase. Therefore, the load corresponding to the displacement of point 2 is the allowable tensile force, and its position on the load curve can be obtained by the vertical green line on the right.

**Figure 10:**
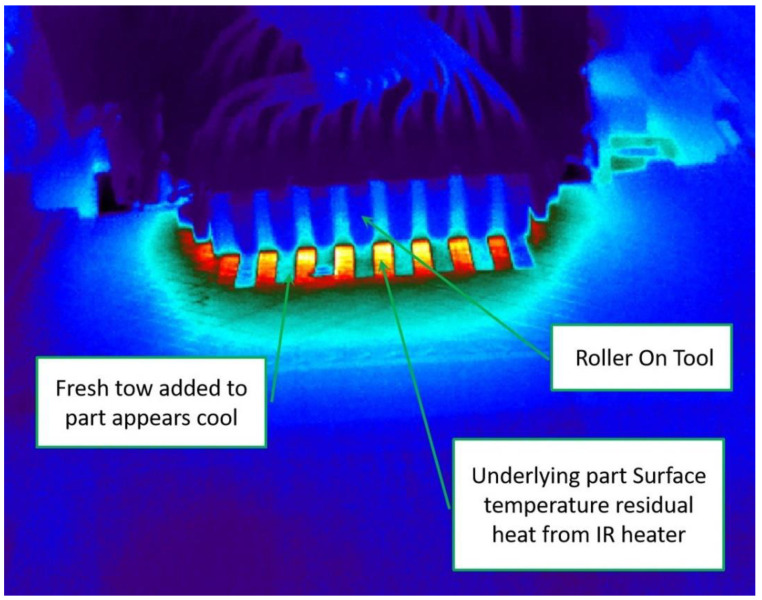
Infrared image of laying temperature test.

To make the probe test results more accurate, the probe test temperature needs to be in line with the actual laying process. In the actual laying process, the prepreg is cooled in the laying head to improve equipment reliability and is heated at the nip point. Figure 10 is an infrared image of a laying temperature test. In this experiment, only odd-numbered prepregs were laid. After laying is completed, the temperature of the new layer is close to room temperature, and the laying temperature of 40 °C is limited to the nip point. In this probe test, the principle of time–temperature equivalence is only used to calculate the pressure-holding time $t_{p}$ corresponding to different laying speeds; that is, it is only used to simulate the bonding process. On the other hand, the separation section is conducted at room temperature, and the test result represents the strength of the layer at room temperature. This just reflects the process in which delamination defects of the prepreg gradually occur under tension after the bonding is formed, so the measured allowable stress is of practical significance.

According to the probe area S, the allowable normal stress $\sigma_{T}$ can be calculated:

(12) $$\sigma_{T}=\frac{F_{s}}{S}$$

**Figure 11:**
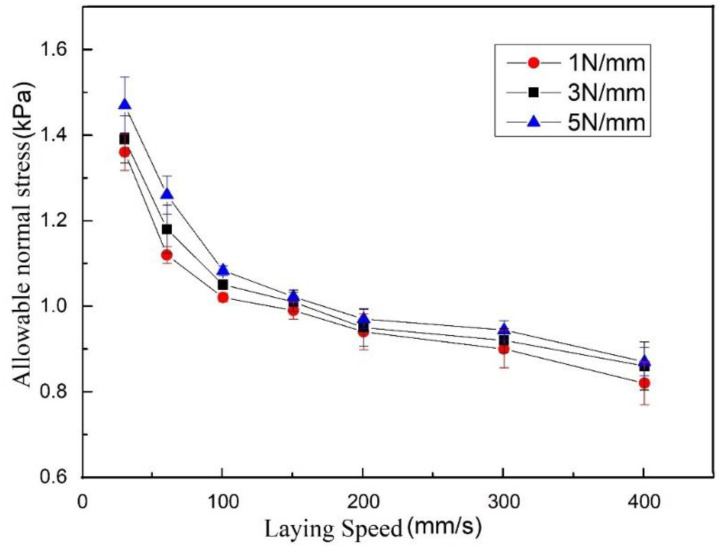
Laying speed/allowable normal stress curve of prepreg/metal laminate.

According to the experimental conditions listed in Section 4.2.1, five experiments are carried out for each experimental condition, and the average value is taken. The following laying speed/allowable normal stress curve can be obtained, as shown in Figure 11.

#### 4.2.3. Allowable Normal Stress Measurement Result of Prepreg/Prepreg Laminate

**Figure 12:**
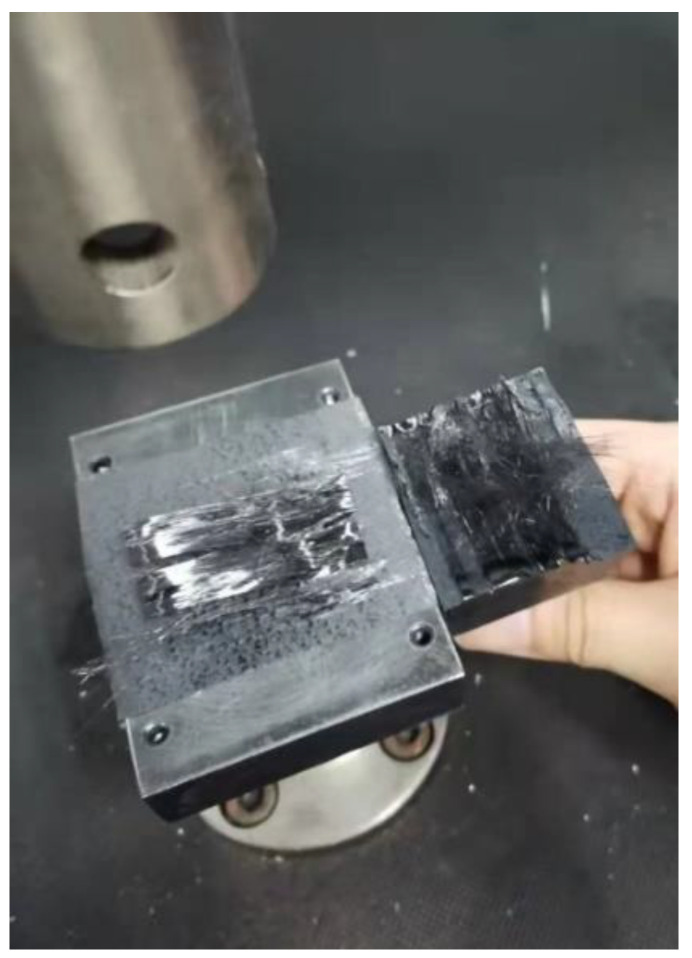
Delamination damage of prepreg in the low-speed experiment.

This experimental method is the same as the allowable stress measurement of the prepreg/metal laminate, but the upper and lower probe surfaces are both glued with prepreg, and the two layers of prepreg are placed in the same direction. In addition, the laying speed in this experiment started from 100 mm/s instead of 30 mm/s. This is because it was discovered during the experiment that when the pressure holding time is too long, serious prepreg delamination damage will occur, as shown in Figure 12. This shows that for the prepreg/prepreg laminate, although the lower laying speed can increase the interlayer bonding strength, it will cause the interlayer bonding strength to be greater than the intralayer strength. If a layup error occurs at this time, the wrong laminate cannot be removed without damage, which does not meet the requirements of AFP. Therefore, it is of greater practical significance to investigate high-speed laying without delamination damage.

**Figure 13:**
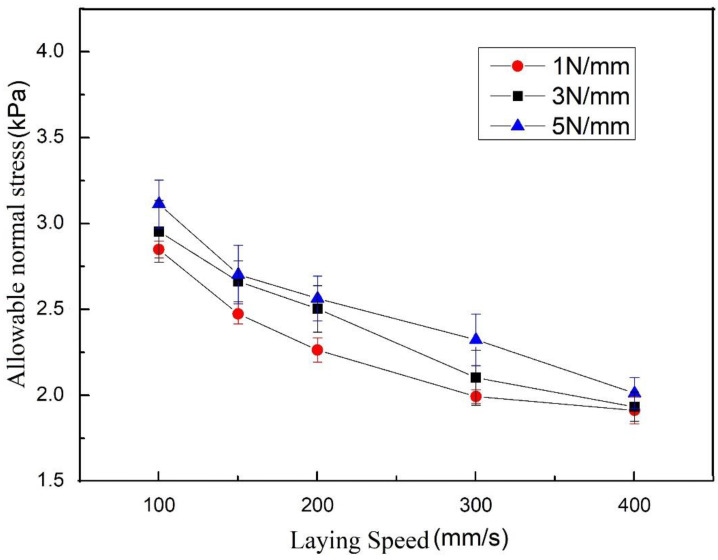
Laying speed/allowable normal stress curve of prepreg/prepreg laminate.

**Figure 14:**
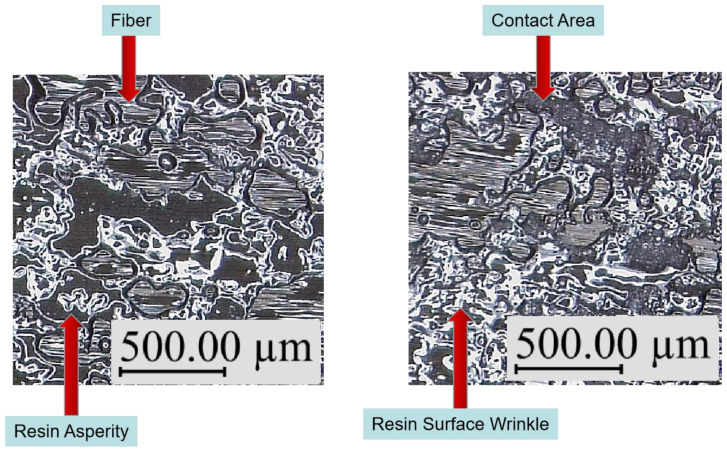
The contrast of prepreg raw surface (**left**) and separated probe test sample (**right**).

According to the experimental conditions listed in Section 4.2.1, five experiments are carried out for each experimental condition, and the average value is taken. Figure 13 shows the obtained laying speed/allowable normal stress curve:

Figure 14 shows the comparison between the surface of the prepreg raw material (left) and the surface of the prepreg (right) after the probe test. It can be seen from the figure that the surface of the prepreg is mainly composed of the resin asperity area and fiber area. Comparing the two surface states, it can be seen that gray areas appear on the surface of the resin asperities on the right side, and there are white dots on the surface of these areas. This shows that after the interface is separated, there are damages such as resin filament on the resin surface, and the gray area is the separated bonding area. At the same time, after the probe test, resin asperities seem to have become denser, and the resin surface has wrinkles, which indicates that the resin asperities have deformed after the prepreg is pressed, thereby expanding the contact area. However, the above analysis is only qualitative. Because the surface morphology of the prepreg is very complicated, it is difficult to determine the contact area through topography. In addition, the allowable normal stress in this study is measured when the sample is not completely separated, so the morphology of the separated sample is not of obvious significance to the research-related mechanism, and the interface of the unseparated sample is difficult to observe using microscopy. For this reason, the result analysis section of this study uses a macroscopic numerical method to analyze the changing trend of allowable normal stress with the process parameters.

## 5. Result Analysis

### 5.1. Physical Meaning of Allowable Normal Stress

The concept of allowable normal stress is proposed based on the process requirements; that is, if the normal stress between plies reaches the allowable normal stress, the thickness of the ply will exceed the thickness of the raw material, causing defects in the plies. However, some noteworthy phenomena appeared in the experiment, indicating that the allowable normal stress may also have a certain meaning from the perspective of the physical process.

**Figure 15:**
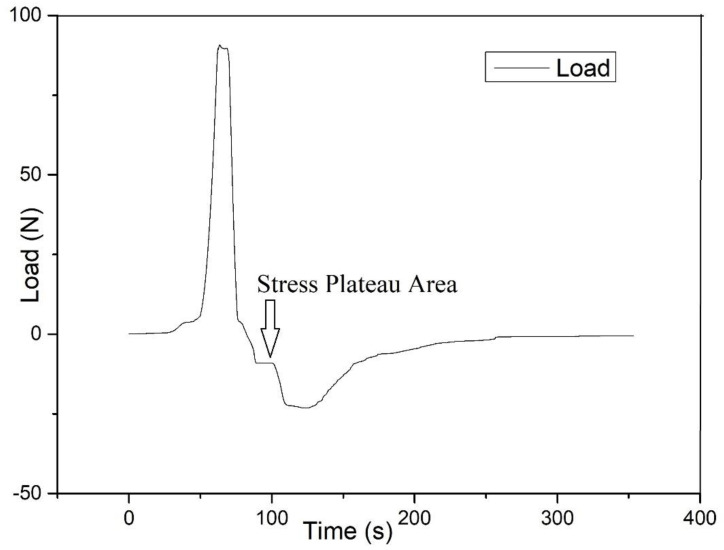
Stress plateau area.

In the probe test, it was found that during the separation process, a plateau interval with a small slope appears in the time/load curve, and the measured allowable normal stresses nearly all lie in this interval, as shown in Figure 15. In this interval, the displacement increases, but the load is almost unchanged, which means the elastic modulus of resin seems to be decreasing. Because the prepreg resin is in a rubbery state at the experimental temperature, this may mean that this interval is just the transition stage from the elastic deformation of the resin to the plastic deformation.

**Figure 16:**
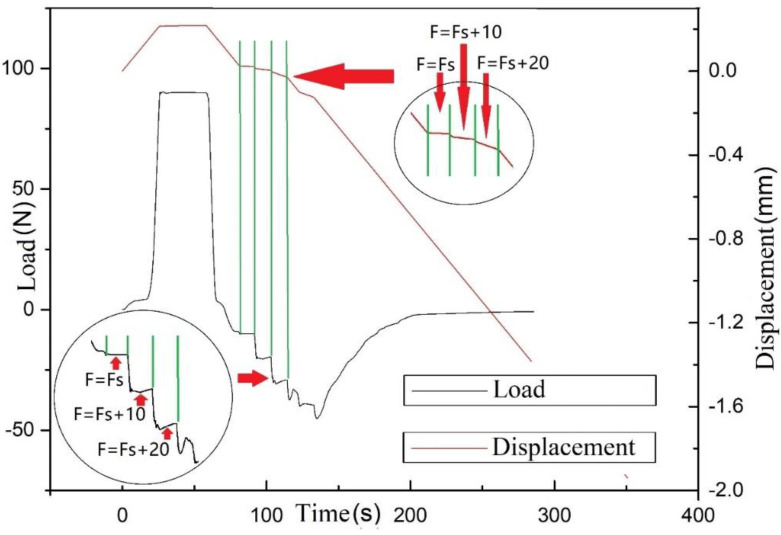
Constant tension test curve.

To verify the above conjecture, a constant force separation experiment of the sample was carried out. The experimental method used was to prepare a sample under the process conditions of known allowable normal stress, and then perform constant stretching with different tensile forces to observe the tensile force and displacement curves. If the tensile force and displacement can be maintained stably, it means that the tensile force is still within the elastic range of the resin. If not, it indicates that the resin has undergone plastic deformation. The curve obtained from the experiment is shown in Figure 16.

The first load plateau in Figure 16 is the measured allowable normal stress. On this basis, the load is increased by 10 N stepwise for experiments. When the stress is less than the allowable normal stress, the tensile force can be maintained stably, and the displacement is almost unchanged. When the stress is greater than the allowable normal stress, the load curve tends to drift away from constant, which means that the tension force is stretching the sample apart and it takes time for the universal testing machine to follow the induced displacement to keep the tensile forces constant. On the other hand, the displacement curve is no longer stable at each plateau and the creep effect appears. When the tensile force reaches $F_{s}+20(N)$ or more, the sample separates quickly and the tensile force cannot be maintained so that the displacement quickly moves in the negative direction, and the load tends to zero. This further shows that the allowable normal stress is close to the elastic limit of prepreg resin. At the same time, this also shows that, during the AFP process, if the stress between layers is greater than the allowable normal stress, the distance between layers will tend to expand and defects are likely to occur.

### 5.2. The Relationship between Allowable Normal Stress and Laying Speed

It can be seen from Figure 11 and Figure 13 that the allowable normal stress increases with the increase in the laying pressure, which shows that as the laying pressure increases, the bonding area of the resin asperities between the prepreg layers increases. In addition, under the same process parameters, the maximum allowable normal stress of the prepreg/prepreg laminate is higher than that of the prepreg/metal laminate, which indicates that the bonding strength between the prepregs is greater than that between the prepreg and metal. The above conclusions are consistent with the existing research results, so they will not be discussed in detail.

This research focuses on the relationship between allowable normal stress and laying speed. As mentioned in Section 2.3, the bonding strength between layers is proportional to the product of the interface resin’s tight bonding rate $D_{ic}$ and unit area strength $\sigma_{i}$. The former is determined by the resin flow, and the latter is determined by the degree of molecular diffusion at the interface. According to Equations (6) and (9), to further understand the determinants of allowable normal stress and quantitatively analyze its relationship with laying speed, the laying speed/allowable normal stress curve can be transformed into a logarithmic coordinate system, a straight-line fitting can be performed, and the slope of the curve can be analyzed.

**Figure 17:**
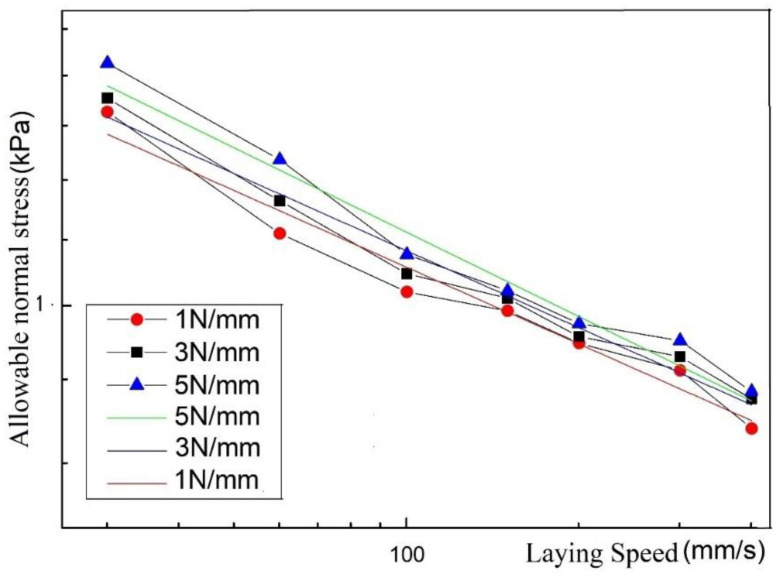
Laying speed/allowable normal stress logarithmic curve of prepreg/metal laminate.

Performing the above transformation on the laying speed/allowable normal stress curve of the prepreg/metal laminate, the following curve can be obtained:

It can be seen from Figure 17 that the laying speed/allowable normal stress logarithmic curve can be approximated to a straight line with a slope close to −1/5 after being fitted with a straight line. When the compactor pressing force is 1 N/mm, 3 N/mm, and 5 N/mm, the corresponding slopes are −0.1766, −0.1775, and −0.1943, respectively. This result is close to the theoretical derivation of Equation (6). This proves that because there is no molecular diffusion on either side of the interface in the prepreg/metal layer, the allowable stress is mainly determined by the deformation of the resin. Under a fixed laying pressure, the allowable stress and laying speed are close to the following relationship:

(13) $$\sigma_{T}\propto V^{-\frac{1}{5}}$$

**Figure 18:**
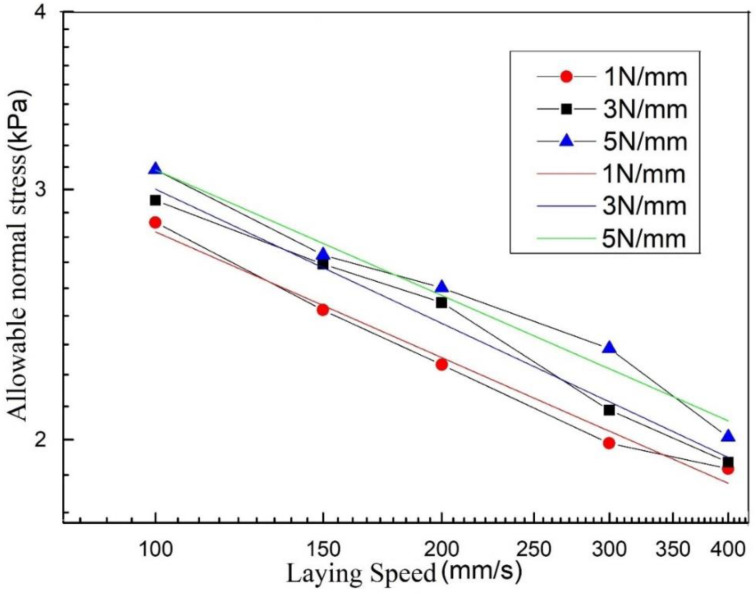
Laying speed/allowable normal stress logarithmic curve of prepreg/prepreg laminate.

Performing the above transformation on the laying speed/allowable normal stress curve of the prepreg/prepreg laminate, the following curve can be obtained:

It can be seen from Figure 18 that after the laying speed/allowable normal stress logarithmic curve is fitted with a straight line, the slopes of curves obtained at the compactor pressing forces of 1 N/mm, 3 N/mm, and 5 N/mm are −0.2938, −0.3134, and −0.2931, respectively. This result is between −0.2 and −0.45. This proves that the interpenetration of molecules on both sides of the interface exists in the prepreg/prepreg laminate, and the allowable stress is determined by two effects, namely resin deformation and molecular diffusion. As the holding time increases, molecular diffusion continues to occur on the newly formed interface, so that the allowable normal stress of the prepreg/prepreg ply increases faster than the prepreg/metal laminate with the increase in pressure holding time.

## 6. Application of Experimental Results in Production

The results of this study have guiding significance for the automatic placement of parts with complex surface features. In the figure below, an eight-fiber robotic AFP machine designed by the author of this article is manufacturing a part with a complex curved surface (the single fiber width W is 6.35 mm).

**Figure 19:**
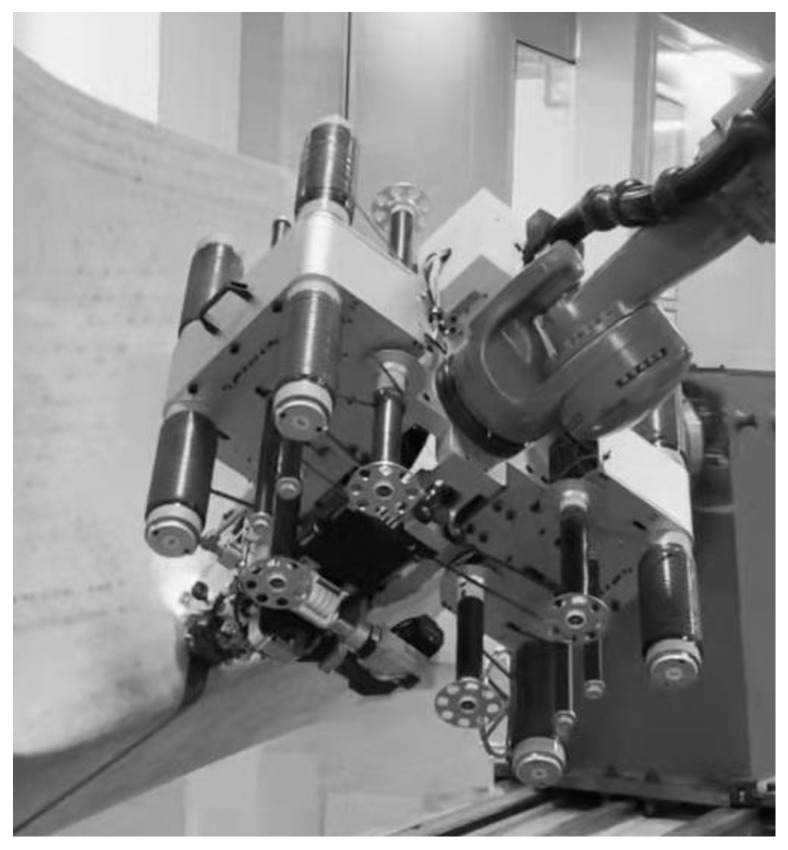
Engineering case: a part with a complex curved surface made by a robotic AFP machine.

The part shown in Figure 19 has a complex body with multiple concave surfaces, convex surfaces, and ridgelines, and the radius of the concave surface varies from 350 mm to 400 mm. For this type of part, the mold surface that the same laying path passes through often has both convex and concave features, which brings great challenges in ensuring the laying quality. The main problem is that when the laying path passes through the ridgeline area, the flexible compactor cannot press the prepreg on the ridgeline all the way. At this time, the fiber tension needs to be maintained at a large value since it is used to achieve bonding in a manner similar to filament winding. However, as mentioned earlier, when the laying path passes through a concave surface, excessive fiber tension will cause bridging defects. Therefore, it is necessary to switch the fiber tension in real-time according to the geometric characteristics of the path.

**Figure 20:**
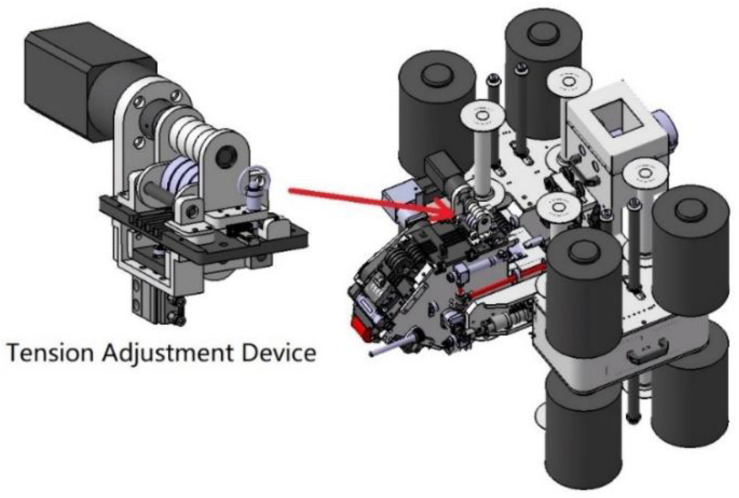
AFP head with real-time tension adjustment device.

In the thermosetting automatic placement process, the main function of the tension of the prepreg is to remove the backing film of the prepreg by winding it on a passive roller. Generally, the fiber tension is maintained at about 5 N. To achieve the goal of laying on complex curved surfaces, a real-time tension adjustment device is specially integrated on the AFP head, as shown in Figure 20.

**Figure 21:**
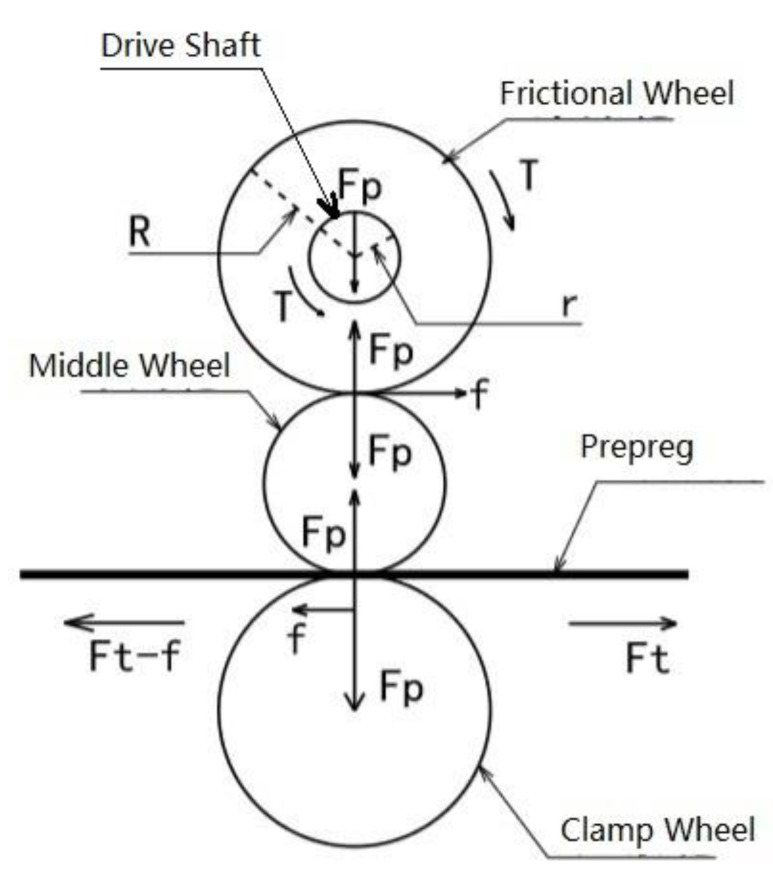
Free body diagram of the real-time tension adjustment device.

The real-time tension adjustment device is mainly composed of a drive shaft, frictional wheels, middle wheels, and clamp wheels. Figure 21 is the free body diagram of a real-time tension adjustment device. During the laying process, the prepreg advances from right to left in Figure 21. An air cylinder is used to apply force to the clamp wheel, and the prepreg is clamped between the clamp wheel and the middle wheel. The middle wheel can float up and down freely, and the clamping force is transmitted to the frictional wheel through the middle wheel. The frictional wheel is placed on a motor-driven shaft and slides against it. The shaft rotates counterclockwise at a speed greater than the maximum fiber laying speed.

Suppose the friction coefficient between the driveshaft and the frictional wheel is μ, the prepreg tension is $F_{t}$, and the clamping force from the clamp wheel to the middle wheel is $F_{P}$. It can be seen from Figure 21 that the clamp force is transmitted to the drive shaft by the frictional wheel. Since the drive shaft rotates counterclockwise and the speed is greater than the linear speed of the prepreg, a sliding frictional force will be generated between the driveshaft and the frictional wheel, the magnitude of which is $F_{P}\cdot \mu$. This frictional force will generate a driving torque $T=F_{P}\cdot \mu \cdot r$ to the frictional wheel. When the prepreg is laid at a uniform speed, the frictional wheel torque is balanced, so the middle wheel has a friction force $f=\frac{T}{R}=\frac{F_{P}\cdot \mu \cdot r}{R}$ on the frictional wheel. Since the maximum static friction force between the frictional wheel and the middle wheel is $F_{P}\cdot \mu$, which is significantly greater than f, slip occurs between the frictional wheel and the drive shaft instead of between the frictional wheel and the middle wheel. Since the middle wheel also rotates at a uniform speed at this time, the driving force of the middle wheel on the prepreg is also f. Therefore, after passing through the device, the tension of the prepreg will become:

(14) $$F_{t}^{\prime }=F_{t}-f=F_{t}-\frac{F_{p}\cdot \mu \cdot r}{R}$$

When $F_{t}>f$, the movement state of the prepreg after passing the tension adjustment device remains unchanged, but the tension decreases. By adjusting the clamping force $F_{P}$, that is, adjusting the air pressure of the clamp cylinder, the tow tension can be adjusted freely. According to experiments, this device can freely adjust the tension of the prepreg between 1.5 and 5 N.

**Figure 22:**
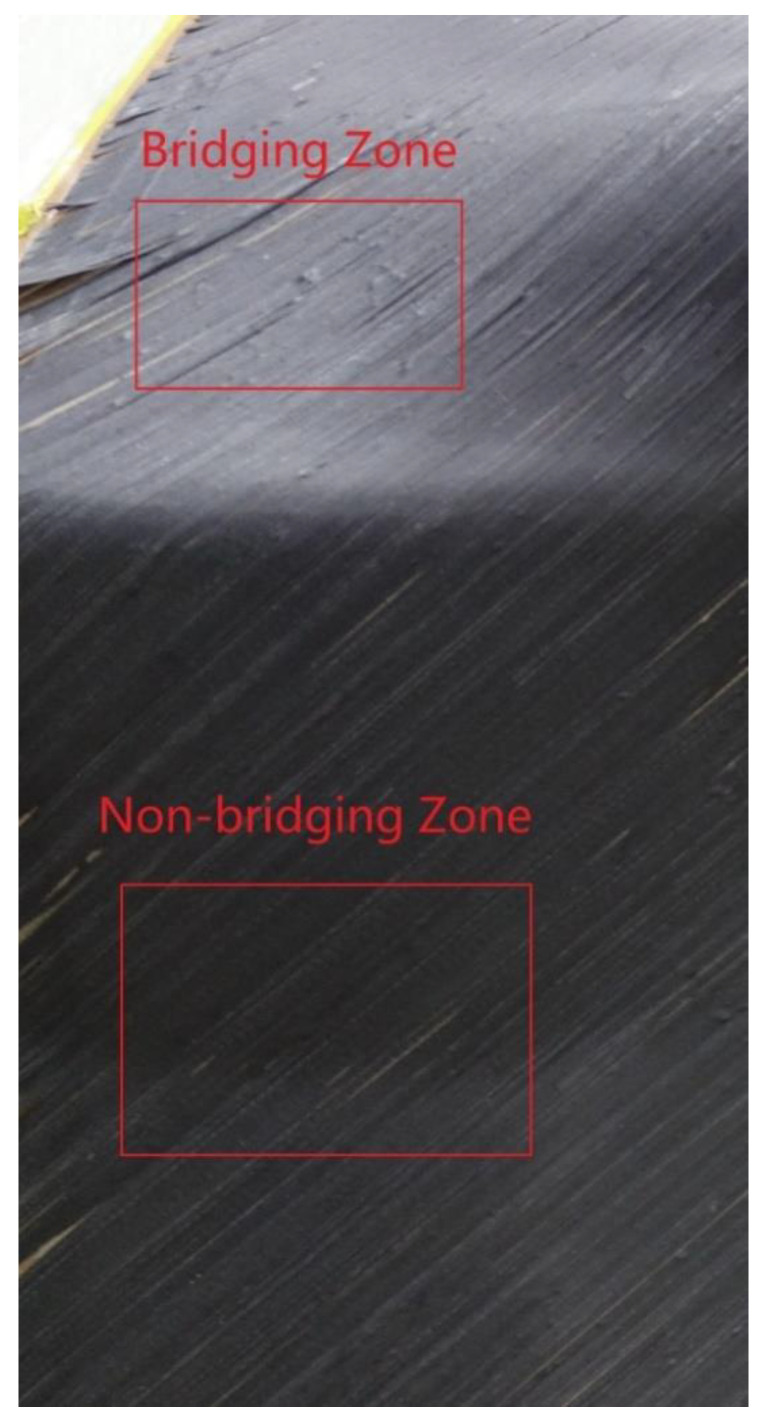
The surface state of the first layer is made by AFP: with and without tension adjustment.

In this project, the user has special requirements for process parameters, and the laying speed is limited to 100 mm/s. According to the allowable stress value measured in Section 4.2.2, when the compactor pressing force is 1 N/mm, 3 N/mm and 5 N/mm, and the laying speed is 100 mm/s, the allowable normal stresses between the prepreg and the mold are 1.02 kPa, 1.05 kPa, 1.08 kPa, respectively. As the laying pressure increases, the allowable normal stress changes very little. The compactor pressing force selected in this project is 1 N/mm. Since the radius of the concave surface of the mold is between 350 mm and 400 mm, according to Equation (2), if the prepreg tension is 5 N, in order to prevent bridging defects, the allowable normal stress needs to reach $\frac{5\;N}{0.35\;m\;\times \;1000\;\times \;0.00635\;m}=2.24\;\mathrm{kPa}$ or more. This shows that when the prepreg tension is maintained at 5 N without using the tension adjustment device, bridging defects will occur when passing through the concave surface. However, if the tension of the prepreg is adjusted to 1.5 N by the tension adjusting device, the allowable normal stress only needs to reach $\frac{1.5\;N}{0.35\;m\;\times \;1000\;\times \;0.00635\;\mathrm{mm}}=0.67\;\mathrm{kPa}$ to prevent bridging defects from occurring. Research has proved this conjecture. Figure 22 shows the surface state of the first layer on the mold. The tension adjustment device is not used in the upper red frame area, and the prepreg tension is maintained around 5 N, so bridging defects occur. The tension adjustment device is used in the lower red frame area, and the tension of the prepreg is maintained at 1.5–2 N. Bridging defects have not occurred.

**Figure 23:**
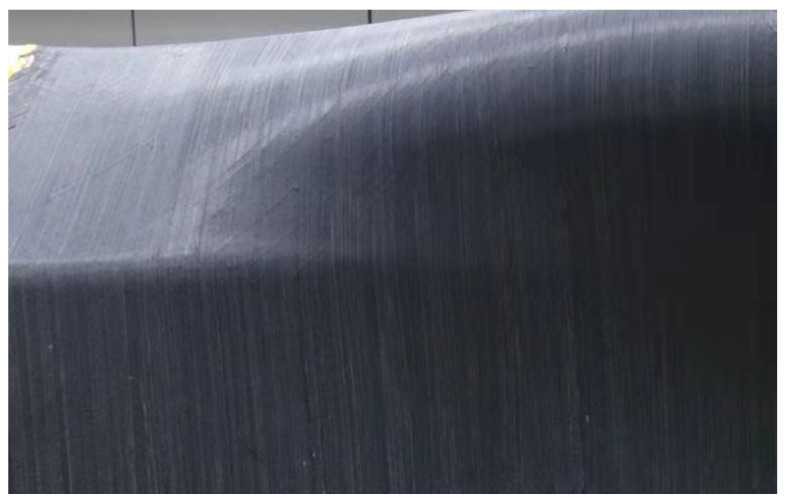
The surface state of the second layer is made by AFP: without tension adjustment.

Another problem is the selection of process parameters in this project is whether it is necessary to use a tension adjustment device to reduce tension in addition to the first layer. Adjusting the tension according to the path curvature will increase the workload of offline programming, and the tension should not be reduced when the laying path passes through the ridge area. Therefore, if bridging defects do not occur, tension adjustment devices should not be used. According to the measurement results in Section 4.2.3, when the compactor pressing force is 1 N/mm, 3 N/mm and 5 N/mm and the laying speed is 100 mm/s, the allowable normal stress of the prepreg/prepreg interface is 2.85 kPa, 2.95 kPa, and 3.11 kPa, respectively. According to the preceding information, when the allowable normal stress is greater than 2.24 kPa, the 5 N prepreg tension will not cause bridging defects. Therefore, the tension adjustment device was not used when the second layer was laid. Figure 23 shows the surface state of the second layer. There is no bridging defect on the surface of the workpiece, which also proves the correctness of the measurement results.

## 7. Conclusions

To characterize the conditions under which prepreg layer separation occurs during the AFP process, this study puts forward the concept of allowable normal stress. According to the principle of volume conservation, when the thickness of the ply is greater than the thickness of the raw material under normal tension of the prepreg, defects such as voids will occur between the layers. Therefore, the normal stress measured at this time is defined as the allowable normal stress to determine the conditions for the occurrence of interlayer defects.

By measuring the time–temperature equivalent coefficient of the M21C prepreg from room temperature to AFP process temperature, probe tests are conducted to simulate the high-temperature and high-speed AFP process at a low temperature and low speed, and the allowable normal stress under various working conditions is measured. Through research, it has been found that for the prepreg/metal laminate, the allowable normal stress is dominated by resin deformation, and its magnitude is approximately proportional to the −1/5th power of the laying speed. For the prepreg/prepreg laminate, the allowable normal stress is determined by the resin deformation and molecular diffusion, and the experiment shows that it is approximately proportional to the −0.3 power of the laying speed.

In the study, it was found that during the constant-speed separation of the ply, near the allowable normal stress, the tensile force appears to reach a plateau, and it is almost constant with the increase in displacement. At the same time, if the allowable normal stress equivalent tensile force is applied to the ply, the thickness of the ply can be maintained, but if the tensile force is increased, the creep phenomenon will occur, and the thickness and stress cannot be maintained. This may indicate that the allowable normal stress is just in the transition interval of the prepreg resin from the elastic zone to the plastic deformation zone, but the mechanism of this phenomenon still needs further study.

This research is of instructive significance for the automatic placement of workpieces with complex surface features. In the engineering case cited in the article, the engineers adjusted the prepreg tension in real-time according to the laying speed and allowable normal stress to prevent bridging effects. The correctness of probe test results is proved by the corresponding surface states of prepreg layers.

## Figures and Tables

**Table 1:** Pressure holding time of 1 N/mm laying pressure probe test.

V **(mm/s)**	30	60	100	150	200	300	400
$t_{p}$ **(s)**	32.05	16.02	9.60	6.41	4.81	3.20	2.40

**Table 2:** Pressure holding time of 3 N/mm laying pressure probe test.

V **(mm/s)**	30	60	100	150	200	300	400
$t_{p}$ **(s)**	44.87	22.43	13.46	8.97	6.73	4.49	3.37

**Table 3:** Pressure holding time of 5 N/mm laying pressure probe test.

V **(mm/s)**	30	60	100	150	200	300	400
$t_{p}$ **(s)**	57.69	28.84	17.31	11.54	8.65	5.77	4.33

## Data Availability

The data presented in this study are available on request from the corresponding author.
